# Host-Diet Effect on the Metabolism of *Bifidobacterium*

**DOI:** 10.3390/genes12040609

**Published:** 2021-04-20

**Authors:** Maria Satti, Monica Modesto, Akihito Endo, Takeshi Kawashima, Paola Mattarelli, Masanori Arita

**Affiliations:** 1 Department of Genetics, SOKENDAI University, Mishima 411-8540, Japan; msatti@nig.ac.jp; 2 Department of Agricultural and Food Sciences, University of Bologna, 40127 Bologna, Italy; monica.modesto@unibo.it; 3 Department of Food, Aroma and Cosmetic Chemistry, Tokyo University of Agriculture, Hokkaido 099-2493, Japan; a3endou@nodai.ac.jp; 4 Bioinformation and DDBJ Center, National Institute of Genetics, Mishima 411-8540, Japan; takeshik@nig.ac.jp; 5 RIKEN Center for Sustainable Resource Science, Yokohama 230-0045, Japan

**Keywords:** *Bifidobacterium*, evolution, glycoside hydrolase, phylogenetics, comparative genomics

## Abstract

*Bifidobacterium* has a diverse host range and shows several beneficial properties to the hosts. Many species should have co-evolved with their hosts, but the phylogeny of *Bifidobacterium* is dissimilar to that of host animals. The discrepancy could be linked to the niche-specific evolution due to hosts’ dietary carbohydrates. We investigated the relationship between bifidobacteria and their host diet using a comparative genomics approach. Since carbohydrates are the main class of nutrients for bifidobacterial growth, we examined the distribution of carbohydrate-active enzymes, in particular glycoside hydrolases (GHs) that metabolize unique oligosaccharides. When bifidobacterial species are classified by their distribution of GH genes, five groups arose according to their hosts’ feeding behavior. The distribution of GH genes was only weakly associated with the phylogeny of the host animals or with genomic features such as genome size. Thus, the hosts’ dietary pattern is the key determinant of the distribution and evolution of GH genes.

## 1. Introduction

Commensal gut bacteria are environment-specific and evolve together with their hosts. The genus *Bifidobacterium* is a widespread and abundant genus belonging to phylum Actinobacteria and is mainly distributed in the intestinal environments of various animals, from insects to mammals [1,2,3,4,5,6]. They have been considered beneficial microorganism to host health. With respect to humans, they are the first colonizers of gut microbiota; a vertical transmission from mother to offspring in humans but also in other animals plays a fundamental role in bifidobacterial occurrence in the gut microbiota. Moreover, colonization of bifidobacteria is modulated by “indigestible” carbohydrates, such as oligosaccharides derived from breastmilk in mammals and plants. These compounds together with the physiology of the host are important drivers of bifidobacterial host co-evolution. It has been shown that certain bifidobacterial species are both host- and niche-specific. Examples of host-specific species are *Bifidobacterium breve* for humans, *Bifidobacterium rousetti* for bat and *Bifidobacterium reuteri* for marmoset [7,8]. On the other hand, there are some species with cosmopolitan lifestyle such as *Bifidobacterium longum*, isolated from humans and animals, and *Bifidobacterium animalis* and *Bifidobacterium pseudolongum*, isolated from different animal species. Since whole genomes are available for many *Bifidobacterium* strains belonging to different species, several genome-scale analyses revealed the acquisition of specific genes, allowing their host specificity [9].

The genomic reservoir of the genus shows an open pan-genome, harboring a large number of strain-specific genes. The genome composition of host-specific strains shows weak association with the phylogeny of their host animals, especially in terms of accessory genes for amino acid production and carbohydrate degradation [10]. Notably, bee-derived species cluster themselves in a deep branch with small genome sizes [11]. Despite multiple attempts, however, identification of host specificity and elucidation of its mechanism has remained unclear from the whole genome analyses.

In this study, we focus on the relationship between host diets and bacterial glycoside hydrolases (GHs) to investigate the evolutionary relationship between bifidobacteria and host animals. To identify this relationship, bifidobacterial species were classified into 13 different groups based on their host dietary patterns. A comparative analysis approach was used to inspect the genomic features such as genome size and GH gene content among the dietary groups. The phylogenetic relationship among the species was also assessed and the phylogenetic signal for the GH content was calculated. Our comparative analysis provides insight into bifidobacterial adaptation to ecological niches.

## 2. Materials and Methods

### 2.1. Genomic Data and Annotations

For the genus-level classification, the type strain data of the 84 recognized *Bifidobacterium* taxa with 76 species and 8 subspecies (*Bifidobacterium animalis* subsp. *lactis*, *B. longum* subsp. *infantis*, *B. longum* subsp. *suis*, *Bifidobacterium catenulatum* subsp. *kashiwanohense*, *B. pseudolongum* subsp. *globosum*, *Bifidobacterium pullorum* subsp. *gallinarum*, *B. pullorum* subsp. *saeculare*, *Bifidobacterium thermacidophilum* subsp. *thermacidophilum*) were used (Supplementary Table S1). For the multi-host analysis, 66 strains from hosts with varying feeding behavior were used (Supplementary Table S2). For the analysis on *B. animalis* subsp. *lactis*, 45 strains were used (Supplementary Table S3).

Genomic sequences were collected from the NCBI Assembly Database and annotated by the DFAST stand-alone software program [12]. Cluster of Orthologous Group (COG) functional annotations were assigned by performing the Reverse Position-Specific BLAST against the NCBI-CDD and by the Perl script “cdd2cog” (https://github.com/aleimba/bac-genomics-scripts/tree/master/cdd2cog; accessed on 29 October 2019). The host and diet information for each strain was collected manually from the NCBI databases and related publications.

### 2.2. Orthologous Gene Clustering

Orthologous gene clustering was performed using the GET_HOMOLOGUES software package [13] (cutoff: E-value 1.0 × 10^−5^, with minimum percentage coverage of 75%) and clusters were detected by the OrthoMCL algorithm [14]. Gene clusters constituting the pan-genome and the core-genome were selected based on the trend of the COG categories. The ratios of COG classes among different set of core genomes (from 100% to 83% core) was compared and an appropriate core was chosen [15].

### 2.3. Identification of Carbohydrate-Active Enzymes

The HMMER search against the dbCAN HMM database was used to determine carbohydrate active enzymes (CAZymes) [16]. The definition of GH families also follows the CAZy database. The standalone version of dbCAN annotation tool was used to determine their annotations.

### 2.4. Selection of the GH Families for Clustering Bifidobacterium Strains

To classify the bacterial strains with their GH distribution, the selection of the GH families is crucial. GH genes are non-essential, and only two families were shared by all the strains, GH3 and GH36 (Supplementary Table S4). On the other hand, out of 72 GH families, 24 families were present in fewer than 5 strains (<5%). To select the GH families that were moderately shared among the strains, we created GH sets that were shared by 100% of 84 taxa, >95% of the taxa, >90%, >85%, and so on (21 sets). Based on each GH set, we performed a hierarchical clustering of bacterial taxa using the distribution of corresponding GH genes and compared results. The GH set of sharing level >20% (Set 17 in Supplementary Table S4) produced the same clustering result as >15% and >10% (Set 18 and Set 19 in Supplementary Table S4), indicating that the classification using 32~42 GH families was stable. Therefore, we selected the threshold of >20% in this analysis.

### 2.5. Phylogenetic Analysis

To infer the phylogenetic relationship among the type strains, the phylogenetic tree based on 362 strict-core proteins was used. The protein alignments were trimmed using trimAL (-automated 1 option) before concatenation [17], and the alignment was constructed using MAFFT version 7.313 [18]. The tree was built using RaxML version 8.27 using PROTGAMMA-BLOSUM62 substitution model and maximum likelihood method [19]. The tree was rooted with *Scardovia inopinata* JCM 12537^T^. The statistical reliability was evaluated by bootstrap analysis of 1000 replicates with the Bootstrap rapid hill climbing algorithm. The tree was visualized using iTOL (https://itol.embl.de/; accessed on 15 November 2019) [20].

### 2.6. Statistical Analysis

Kruskal-Wallis test (significance level of *p* < 0.05) and Dunn’s post hoc test was performed using the R version 3.6.2. Phylogenetic signal for genomic trait of GH content was calculated using the R package “phylosignal” [21]. GH content is defined as the percentage of GH genes in each bifidobacterial type strain.

To measure the strength of the phylogenetic signal (likelihood of shared evolutionary history), we used Blomberg’s K statistic [22]. The K values closer to 1 and 0 indicate strong and weak evolutionary correlation, respectively. To detect the hotspots of autocorrelation, local Moran’s I for each species and local indicator of phylogenetic association (LIPA) were computed.

## 3. Results and Discussion

### 3.1. Host Diet and the Genome Size of Type Strains

The genomic sequences for 84 *Bifidobacterium* type strains (76 species and 8 subspecies) were investigated. The genome size of the strains ranged from 1.63 to 3.25 Mb with an average of 2.43 Mb (SD ± 0.40). The GC content ranged from 50.4 to 66.6% with an average of 60.8%. The orthologous clustering of their coding genes revealed that the pan-genome amounted to 24,181 gene clusters including singletons.

**Figure 1:**
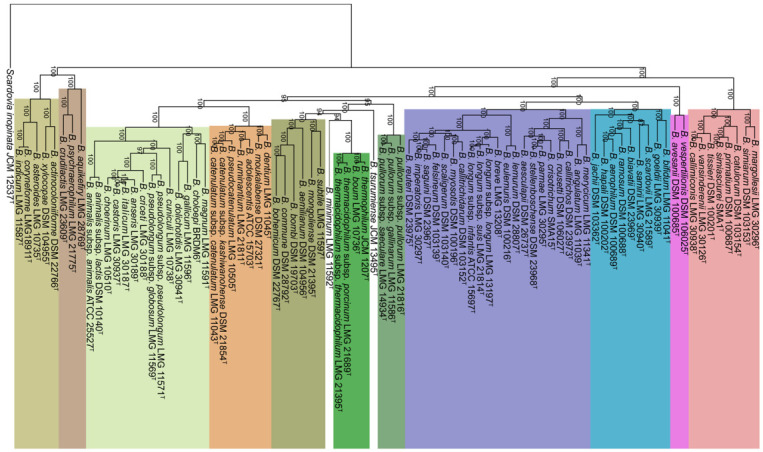
Phylogenetic tree based on concatenated amino acid sequences of 362 core genes of the 84 type strains. Bootstrap percentages of >70 are shown. Eleven phylogenetic groups are highlighted in different colors and the new group is the second rightmost (rose).

The number of clusters shared across ≥80 strains and across all strains were 722 and 362, respectively. The latter strict core was used to construct the phylogenetic tree by concatenating the amino acid sequences of the strict-core genes. In the resulting tree, 10 previously described groups [23] and one additional group were identified. The new group consisted of *Bifidobacterium avesanii* and *Bifidobacterium vespertilionis* (Figure 1). The former strain was isolated from cotton-top tamarin (*Saguinus oedipus*), a new-world monkey in South America feeding mainly on fruits and insects [24]. The latter, *B. vespertilionis*, was isolated from Egyptian fruit-bat (*Rousettus aegyptiacus*) feeding only on the pulp and juice of various fruits [25]. Two strains, *Bifidobacterium tsurumiense* and *Bifidobacterium minimum*, were not included in any cluster.

**Figure 2:**
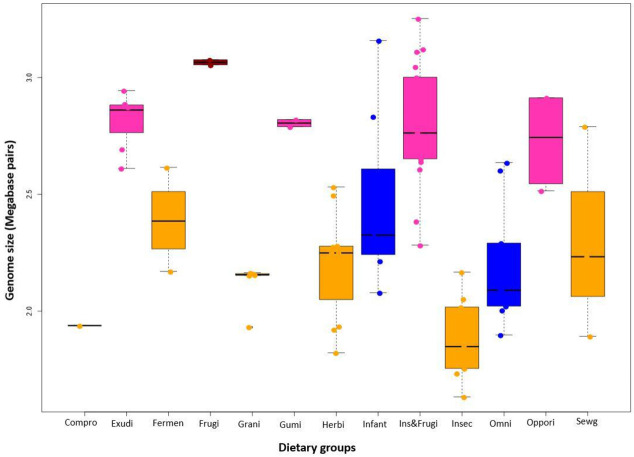
Genome sizes of the strains in each dietary group. The box plot indicates the mean and standard deviation. Compro: Commercial probiotic; Exudi: Exudativore; Fermen: Fermented food; Frugi: Frugivore; Grani: Granivore; Gumi: Gummivore; Herbi: Herbivore; Infant: Infant food; Ins&Frugi: Frugivore eating insects; Insec: Nectarivore, palynivore; Omni: Omnivore; Oppori: Opportunistic omnivore eating fruits, leaves and insects; Sewg: Sewage. Exudi, gumi, and grani eat insects too. The colors in the boxplot show different host groups; Dark red: bats, Pink: monkey/apes, Blue: human/pigs, Yellow: other animals.

To examine the relationship between host diets and the genome sizes, the strains were classified into 13 dietary groups according to the feeding behavior and isolation sources of their hosts (Supplementary Figure S1 and Table S1). Genome sizes differed significantly among the different dietary groups (Kruskal-Wallis chi-squared = 59.101, df = 13, *p*-value = 7.603 × 10^−8^) (Figure 2). Strains from bees showed the smallest genome sizes as previously reported [11]. The genome sizes of strains from herbivores and granivores were similar. Within primate origins, the genome sizes differed between human adults and pigs, feeding on both of plant and animal matter, and monkeys feeding on fruits (frugivore), plant exudates (exudativore), or gums (gummivore). The latter showed a larger genome size while those of human and pig strains were comparable to the sizes in herbivores (leafs) and granivores (grains). Strains from human infants exhibited an intermediate genome size. In all groups, no significant differences were found in the GC content (Supplementary Table S1).

### 3.2. Distribution of Carbohydrate-Active Enzymes

The largest dietary difference between human adults and infants is milk oligosaccharides. Human milk contains diverse non-digestible oligosaccharides, classified into 13 structure series. As we shall see, GH33 (sialidase) is enriched only among strains from human infants, because sialic acid is a characteristic sugar in human milk. To investigate such metabolic correlation comprehensively, all carbohydrate-related genes were first investigated.

**Figure 3:**
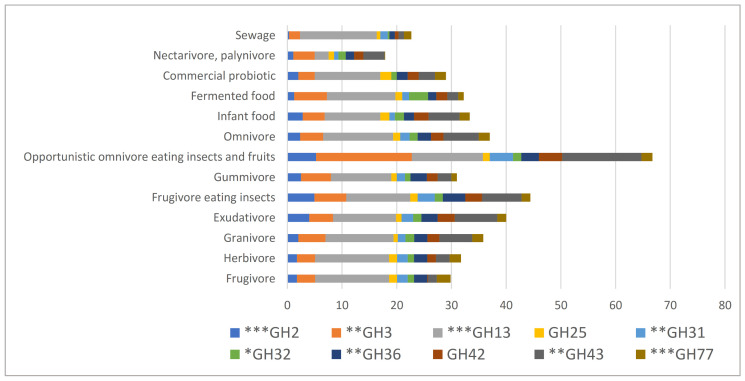
Distribution of the number of glycoside hydrolase genes in the different dietary groups. CAZyme families in >80% of the strains are shown. The significance by Kruskal-Wallis test is shown by asterisks. * *p* < 0.05, ** *p* < 0.01, *** *p* < 0.001.

According to the Carbohydrate Active Enzymes (CAZy) system, each strain possessed from 33 to 166 genes (mean 88; SD ± 29.46). These genes spanned the wide range of CAZy families: 72 GHs (glycoside hydrolases), 17 GTs (glycosyltransferases), 10 CEs (carbohydrate esterases) and 2 PLs (polysaccharide lyases) and 20 CBMs (appended non-catalytic carbohydrate-binding modules). Shared among ≥80% of the strains were 10 GH families (GH2, GH3, GH13, GH25, GH31, GH32, GH36, GH42, GH43, and GH77), 5 GT families (GT2, GT4, GT28, GT35, and GT51), CE10, and CBM48. Among these families, the distribution significantly differed (*p* < 0.01) among hosts of different diets in 7 GH families (GH2, GH3, GH13, GH31, GH36, GH43, and GH77), 3 GT families (GT2, GT4, and GT35), CE10, and CBM48 (Figure 3 and Supplementary Figure S2). Considering the diversity of the gene distribution, we focused on the GH families.

### 3.3. Clustering of Bifidobacterium Species Based on GH Families

**Figure 4:**
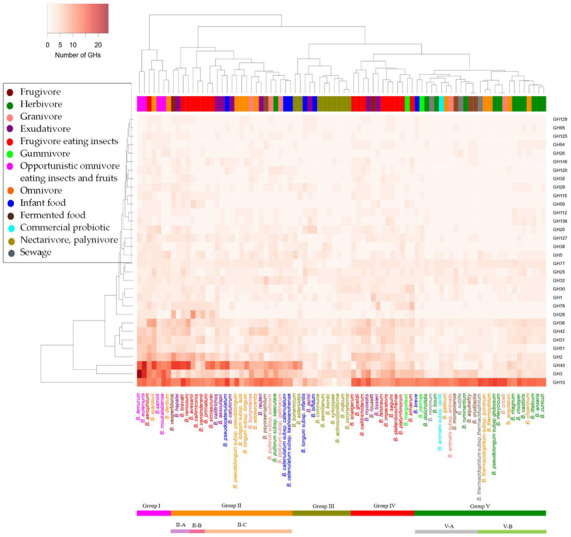
Clustering of bifidobacterial species based on GH family genes. The heatmap shows the gene number for the selected GH families (families present in 20% of the strains). Pink: Group I with the opportunistic omnivores; Orange: Group II with omnivore, herbivore or insectivore; Gold: Group III with nectarivore; Red: Group IV with insectivore and frugivore; Green: Group V with herbivore and mixed diet. Each strain is highlighted with the color of the corresponding diet class.

We next identified key GH families that delineate dietary difference of hosts. The clustering result of GH families became stable when 32 families that were present in >20% of all strains were used (see Methods). The clustering created Group I–V in Figure 4, with the following characteristic families (Table 1).

1. Group I included strains with the largest number of GH genes. This group reflected species from opportunistic omnivore eating insects and fruits. The group had high numbers of GH43 and GH3 genes associated with degradation of complex plant polysaccharides like xylan, arabinan or arabinoxylan. This suggested that these GH genes were adapted to the hosts of mixed diets (omnivore and frugivore).
2. Group II included strains with a high number of GH43 but low GH3. The group included 25 species and was further divided into three: subgroup II-A, -B and -C. The subgroup II-C possessed low numbers of GH2, GH28, GH59 and GH115. The dietary pattern of the hosts varied: omnivore, herbivore, frugivore, insectivore and exudativore.
3. Group III included bee isolates and two infant isolates. This group possessed a very low number of GH13. This result was supported by previous studies where the GHs from the insects clustered separately [26]. GH13 enzymes are involved in degradation of starches and malto-oligosaccharides, and such sugars are usually scarce in diets of bees and infants.
4. Group IV included strains from hosts of insect and fruit diet. This group had the second highest gene counts for GHs after Group I, which suggested that the species from frugivorous hosts possessed more GH genes.
5. Group V included the largest number of strains. This group had the lowest GH gene counts, where many of the GH families were mostly absent (e.g., no GH28, GH38 and GH115). The group was further divided into two subgroups (subgroup V-A and V-B). Subgroup V-B was strains from herbivorous hosts while subgroup V-A included strains from hosts of mixed dietary habits.

In Figure 4, the strains from insectivorous and frugivorous hosts were spread in separate clusters (Group IV and Group II). This discrepancy was attributed to the strains isolated from tamarins, whose diet is mainly insects and fruits but sometimes small amphibians. When the host diet was more complex (e.g., opportunistic omnivore, and frugivore and folivore), more diverse GH families and more genes were found. On the contrary, the strains from hosts with simple feeding habits (e.g., pure herbivore and nectarivore) possessed smaller number of families and genes. A good example was four subspecies of *B. longum*: subsp. *longum*, subsp. *suis*, subsp. *infantis*, and subsp. *suillum*. Of the three subspecies whose genomes were available, the former two belonged to Group II, while subsp. *infantis* belonged to Group III, due to different diets of their hosts. Hosts of the subsp. *longum* and subsp. *suis* are omnivores, while subsp. *infantis* is only seen in human infants. Infants generally consume simple diet, including breast milk and infant formulae, and thus storage of numerous GHs is not essential for the strain.

To test whether the GH contents follow the dietary pattern rather than the phylogeny, we checked the phylogenetic signal for GH genes. The analysis showed weak phylogenetic signal with Bloomberg’s K value closer to 0 (K = 0.448). Phylogenetic correlogram analysis detected nonsignificant autocorrelation above the phylogenetic distance of 0.1 (Supplementary Figure S3a). We also performed the LIPA analysis to identify clades with a high phylogenetic signal. Only two clades (Clade 1: *Bifidobacterium eulemuris* and *Bifidobacterium lemurum*; Clade 2: *Bifidobacterium hapali*, *Bifidobacterium aerophilium*, *Bifidobacterium ramosum*, *Bifidobacterium biavatii*, *Bifidobacterium scardovii*, and *Bifidobacterium samirii*) were detected with significant positive autocorrelation (*p*-value < 0.01) (Supplementary Figure S3b).

### 3.4. Comparison of Bifidobacterium Species from Multiple Host Animals

**Figure 5:**
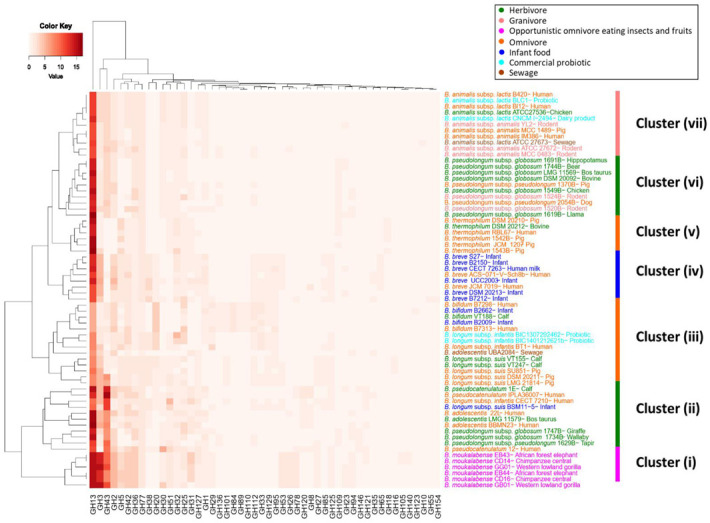
Clustering of 66 strains isolated from different sources based on their GHs. Heatmap displays the number of genes in GH families. Strains were colored according to their host dietary patterns as in the upper box. Strains were clustered in seven major groups: Cluster (**i**) Opportunistic omnivore; Cluster (**ii**) and Cluster (**vi**) Herbivore; Cluster (**iii**) and Cluster (**v**) Omnivore; Cluster (**iv**) Infant food; and Cluster (**vii**) Granivore and Insectivore.

Some species were isolated from multiple host animals with different dietary patterns. To investigate their GHs, we selected 66 strains in 11 different species isolated from different hosts (Supplementary Table S2). Their clustering resulted in seven different groups, from Cluster (i) to Cluster (vii), among which five groups (Cluster (i), (iv), (v), (vi), and (vii)) cleanly corresponded to the species’ phylogeny (*Bifidobacterium moukalabense*, *B. breve*, *Bifidobacterium thermophilum*, *B. pseudolongum*, and *B. animalis*) (Figure 5).

The result suggested that strains within the same species shared similar GH families. Still, we could find characteristic GH families that coincided with host diet patterns. For example, *B. moukalabense* strains from gorilla, chimpanzee, and elephant possessed high numbers of GH families for plant carbohydrates (GH43, GH3, GH13, GH53, GH26 and GH78). *B. thermophilum* from pig, cow, and human lacked GH43 and GH2, and these families hydrolyze plant carbohydrates and milk carbohydrates, respectively (Table 2). *B. bifidum* strains were isolated from infants and calf and possessed high numbers of GH families for milk-origin carbohydrates (GH2, GH20, GH33, GH129 and GH84). Among the milk metabolizing families was GH33 (sialidase), whose abundance is statistically significant in *B. bifidum*, *B. longum* subsp. *infantis*, and *B. breve* only [28].

To further investigate the variation of GH genes within the same species, we selected 45 strains of *B. animalis* subsp. *lactis* from 15 different isolation sources (Supplementary Table S3). Many strains were isolated from humans probably because of extensive use of probiotic strains (re-isolation). The clustering based on GH genes within subsp. *lactis* showed a single large isogenic group with a small, isolated group from dog, pig and food products (Supplementary Figure S4). This result supported that strains in the same species share similar GH patterns. The reason for the large deviation of some strains may be due to an application of unique strains as probiotics for animals. When all available *B. animalis* subsp. *lactis* strains were investigated for their GH genes, the 95% confidence interval for the number of GH genes in each family was never larger than 0.4 (Supplementary Table S5). This indicated that the number of GH genes did not differ much within the same species and justified our approach of using type strains to grasp the overview of metabolic capabilities in *Bifidobacterium.*

## 4. Conclusions

Genome-based features can deepen the understanding of the bacterial adaptation with host. We classified *Bifidobacterium* strains into five groups based on their GH genes, and the key GH families delineated the differences in host diet. The species from hosts having complex dietary habits possessed considerably more GH genes than those having simpler dietary patterns. Furthermore, a weak phylogenetic signal was confirmed for the distribution of GH genes.

In summary, bifidobacteria are adapted to their hosts’ dietary habits, and their GH composition is associated with the diet composition. However, the GH composition within the same species did not match the host diet well. The shuffling speed of GH genes is therefore not faster than the speciation and host adaptation.

## Figures and Tables

**Table 1:** Characteristic GH families in different dietary groups (*p* < 0.05).

Dietary Groups	GH Families	Related Activities in Bifidobacteria [27]
Opportunistic omnivore eating insects and fruits and Frugivore eating insects (Group I, Group II-B and Group IV)	GH13	α-1,4-glucosidase, amylopullulanase, sucrose Phosphorylase, α-amylase
	GH3	β-glucosidase, β-hexosaminidase
	GH43	Endo-1,5-α-l-arabinosidase, α-l-arabinofuranosidase, Endo-1,4-β-xylanase, β-1,4-xylosidase
	GH26	Endo-1,4-β-mannosidase
	GH53	Endogalactanase
	GH31	α-xylosidase
	GH78	α-l-rhamnosidase
	CBM67	l-rhamnose binding activity
Frugivore eating insects (Group II-B and Group IV)	GH115	xylan α-1,2-glucuronidase, α-(4-O-methyl)-glucuronidase
	GH28	Galacturan1,4-α-galacturonidase, pectinesterase
Herbivore (Group V-B)	GH94	Cellobiose-phosphorylase
	GH36	α-galactosidase, raffinose synthase
Infant food (Group II-C)	GH33	Sialidase
	GH20	β-hexosaminidase
	GH29	α-l-fucosidase
	GH95	α-l-fucosidase
	GH112	Lacto-N-biosephosphorylase
	GH29	α-l-fucosidase
	GH95	α-l-fucosidase
Nectarivore and Palynivore (Group III)	GH65	α,α-trehalase
	GH13 *	α-1,4-glucosidase, amylopullulanase, sucrose Phosphorylase, α-amylase
	GT20	α,α-trehalose-phosphate synthase
	GT35 *	glycogen or starch phosphorylase
	CBM48 *	appended to GH13 modules
	CE10 *	arylesterase

Asterisks indicate significantly low occurrences.

**Table 2:** Characteristic GH families in the *Bifidobacterium* species with multiple hosts (*p* < 0.05 by Kruskal-Wallis test).

Family	Related Subfamilies	Significantly High	Significantly Low
GH1	β-glucosidase, β-galactosidase	*B. bifidum*	*B. longum* subsp. *suis*
GH2	β-galactosidase	all others	*B. thermophilum*
GH3	β-glucosidase, β-hexosaminidase, β-glucosideglucohydrolase	*B. thermophilum*, *B. bifidum*	*B. moukalabense*
GH5	β-mannosidase, β-glucosidase, β-exoglucanase	*B. moukalabense*	*B. pseudolongum* subsp*. globosum*
GH13	α-1,4-glucosidase, amylopullulanase, sucrose phosphorylase, α-amylase	*B. moukalabense*	*B. bifidum*
GH20	β-hexosaminidase	*B. bifidum*	all others
GH26	Endo-1,4-β-mannosidase	*B. moukalabense*	all others
GH27	α-galactosidase	*B. moukalabense*	all others
GH29	α-L-fucosidase	*B. bifidum*	*B. thermophilum*
GH30	β-d-xylosidase, endo-1,6-β-glucosidase, Glucosylceramidase	all others	*B. thermophilum*
GH31	α-xylosidase	*B. moukalabense*	all others
GH32	β-fructofuranosidase, sucrose-6-phosphatehydrolase	all others	*B. bifidum*
GH33	Sialidase	*B. bifidum*	*B. pseudolongum* subsp*. globosum*
GH36	α-galactosidase, raffinosesynthase	*B. moukalabense*	*B. thermophilum*
GH43	Endo-1,5-α-l-arabinosidase, α-l-arabinofuranosidase, Endo-1,4-β-xylanase, β-1,4-xylosidase	all others	*B. thermophilum*
GH51	α-L-arabinofuranosidase	*B. moukalabense*	*B. bifidum*
GH53	Endogalactanase	*B. moukalabense*	*B. pseudolongum* subsp*. globosum*
GH77	4-α-glucanotransferase	*B. bifidum*	all others
GH78	α-l-rhamnosidase	*B. moukalabense*	all others
GH84	α-l-rhamnosidase	*B. bifidum*	all others
GH85	Endo-β-N-acetylglucosaminidase D	*B. longum* subsp*. suis*	all others
GH89	α-N-acetylglucosaminidase, β-N-hexosaminidase	*B. bifidum*	all others
GH94	Cellobiose-phosphorylase	*B. moukalabense*	all others
GH95	α-l-fucosidase	*B. bifidum*	all others
GH101	endo-α-N-acetylgalactosaminidase	*B. bifidum*	all others
GH109	α-N-acetylgalactosaminidase	*B. pseudolongum* subsp*. globosum*	all others
GH110	Exo-α-galactosidase	*B. bifidum*	all others
GH112	Lacto-N-biosephosphorylase	*B. bifidum*	all others
GH120	β-xylosidase	*B. pseudocatenulatum*	all others
GH121	β-galactosidase	*B. pseudocatenulatum*	all others
GH127	β-l-arabinofuranosidase	*B. moukalabense*	all others

## Data Availability

All genetic data are available from the GenBank/ENA/DDBJ repository. Accession numbers of all strains are summarized in Supplementary files; Tables S1–S3.

## Supplementary Materials

The following are available online at https://www.mdpi.com/article/10.3390/genes12040609/s1. Figure S1: The number of bifidobacterial strains according to the dietary pattern of their respective hosts; Figure S2: The number of CAZyme and GH genes encoded by the strains in each dietary group; Figure S3: Phylogenetic correlogram and the Local Moran’s index for GH content for each type strain; Figure S4: Clustering of *B. animalis* subsp. *lactis* strains isolated from 15 different isolation sources, based on the number of GH genes. Table S1: Genomic features and diet information of all type strains; Table S2: Isolation sources and accession numbers for 66 strains isolated from various hosts; Table S3: Isolation sources and accession numbers for 45 *B. animalis* subsp. *lactis* strains; Table S4: Selection of GH families for clustering. The chosen set is shown in bold; Table S5: The distribution of GH genes in 434 different *Bifidobacterium* strains.
