# Supplementary material for: Host-Diet Effect on the Metabolism of Bifidobacterium
Source: Genes (Basel). 2021 Apr 20;12(4):609. doi: 10.3390/genes12040609 (PMC8074910; doi:10.3390/genes12040609)
Supplement: Supplementary file 1 [file genes-12-00609-s001.zip › Supplementary Figure S1.pdf]

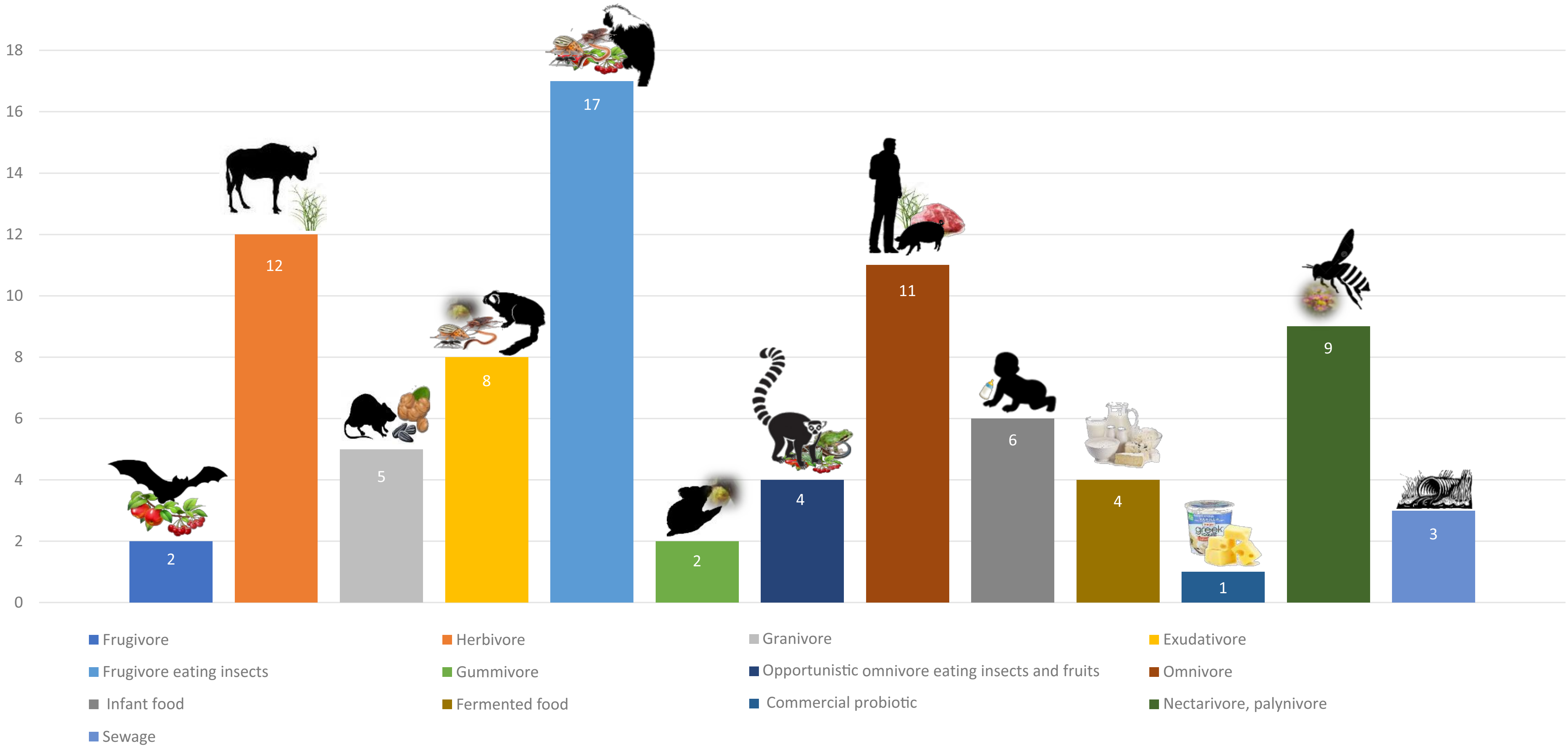

Figure S1. Grouping of bifidobacterial species in accordance with the dietary pattern of their respective host, The chart displays the number of species belonging to each group.
