# Supplementary material for: Host-Diet Effect on the Metabolism of Bifidobacterium
Source: Genes (Basel). 2021 Apr 20;12(4):609. doi: 10.3390/genes12040609 (PMC8074910; doi:10.3390/genes12040609)
Supplement: Supplementary file 1 [file genes-12-00609-s001.zip › Supplementary Figure S2.pdf]

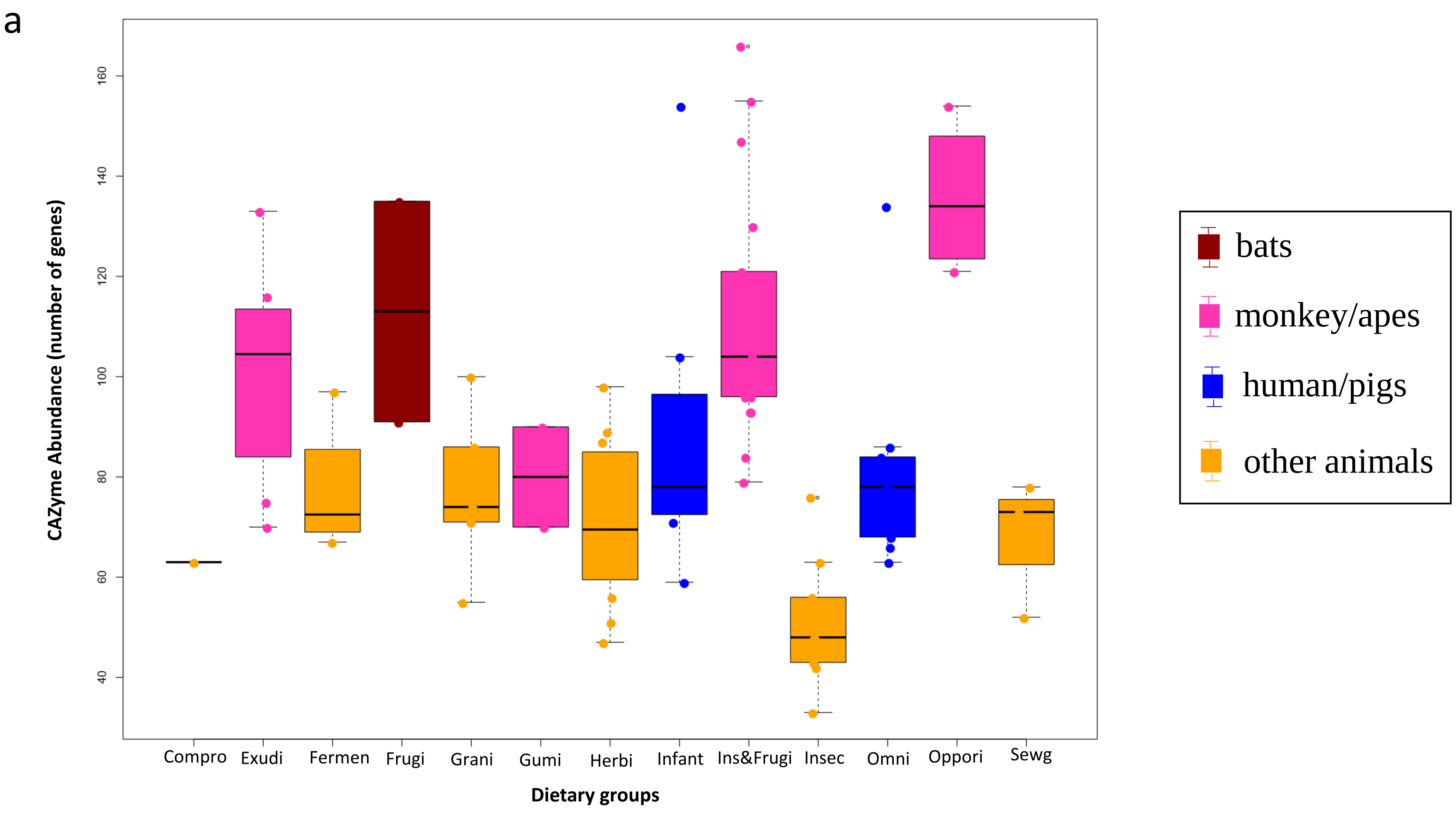

a
CAZyme Abundance (number of genes)
40
60
80
100
120
140
160
Compro
Exudi
Fermen
Frugi
Grani
Gumi
Herbi
Infant
Ins&Frugi
Insec
Omni
Oppori
Sewg
Dietary groups
bats
monkey/apes
human/pigs
other animals

b

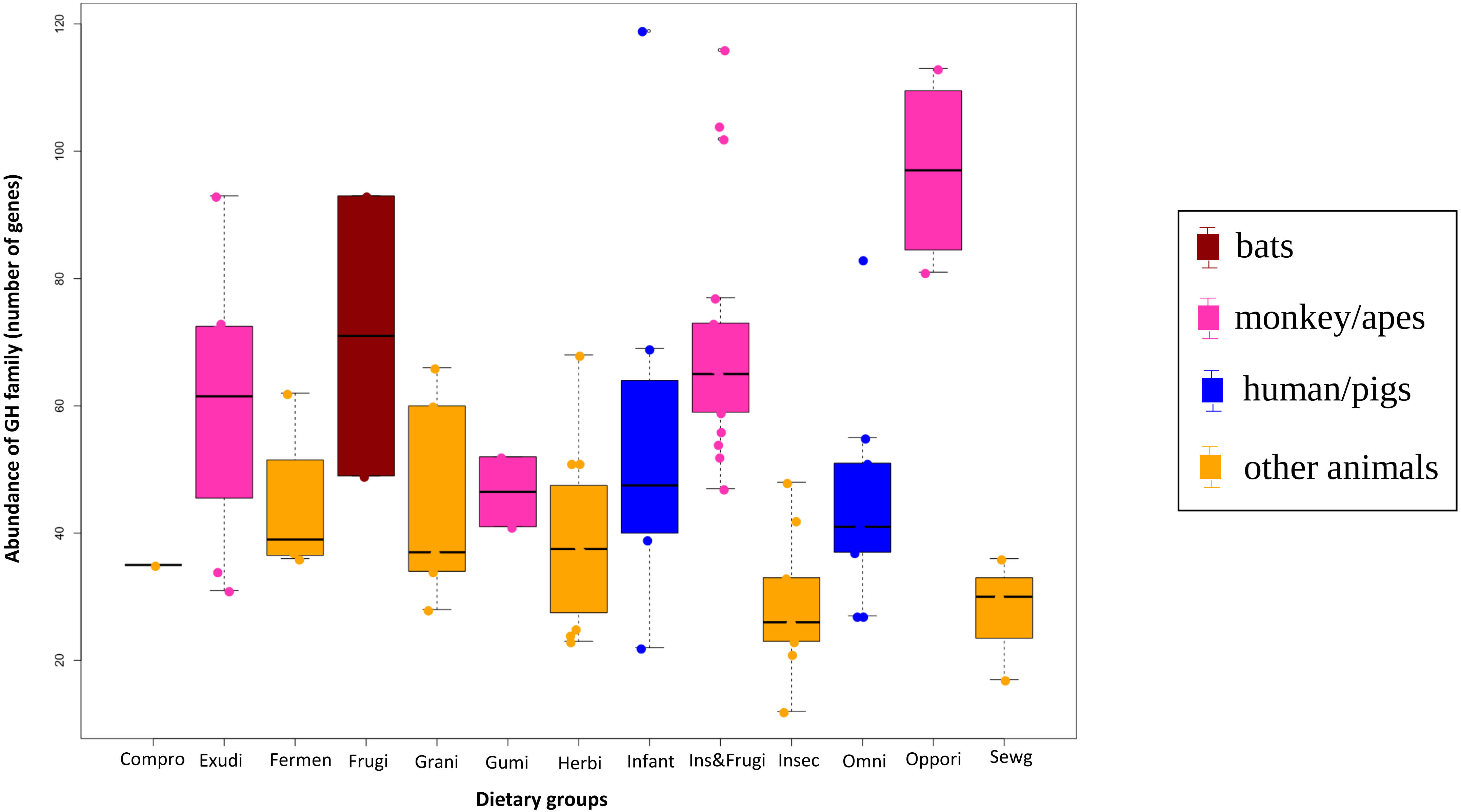

Figure S2. CAZyme families and GHs genes encoded by the strains in each dietary group **(a)** Abundances of the CAZyme families. **(b)** Abundance of genes encoding GHs. Compro: Commercial probiotic; Exudi: Exudativore; Fermen: Fermented food; Frugi: Frugivore; Grani: Granivore; Gumi: Gummivore; Herbi: Herbivore; Infant: Infant food; Ins&Frugi: Frugivore eating insects; Insec: Nectarivore, palynivore; Omni: Omnivore; Oppori: Opportunistic omnivore eating fruits, leaves and insects; Sewg: Sewage.
