# Supplementary material for: Host-Diet Effect on the Metabolism of Bifidobacterium
Source: Genes (Basel). 2021 Apr 20;12(4):609. doi: 10.3390/genes12040609 (PMC8074910; doi:10.3390/genes12040609)
Supplement: Supplementary file 1 [file genes-12-00609-s001.zip › Supplementary Figure S3.pdf]

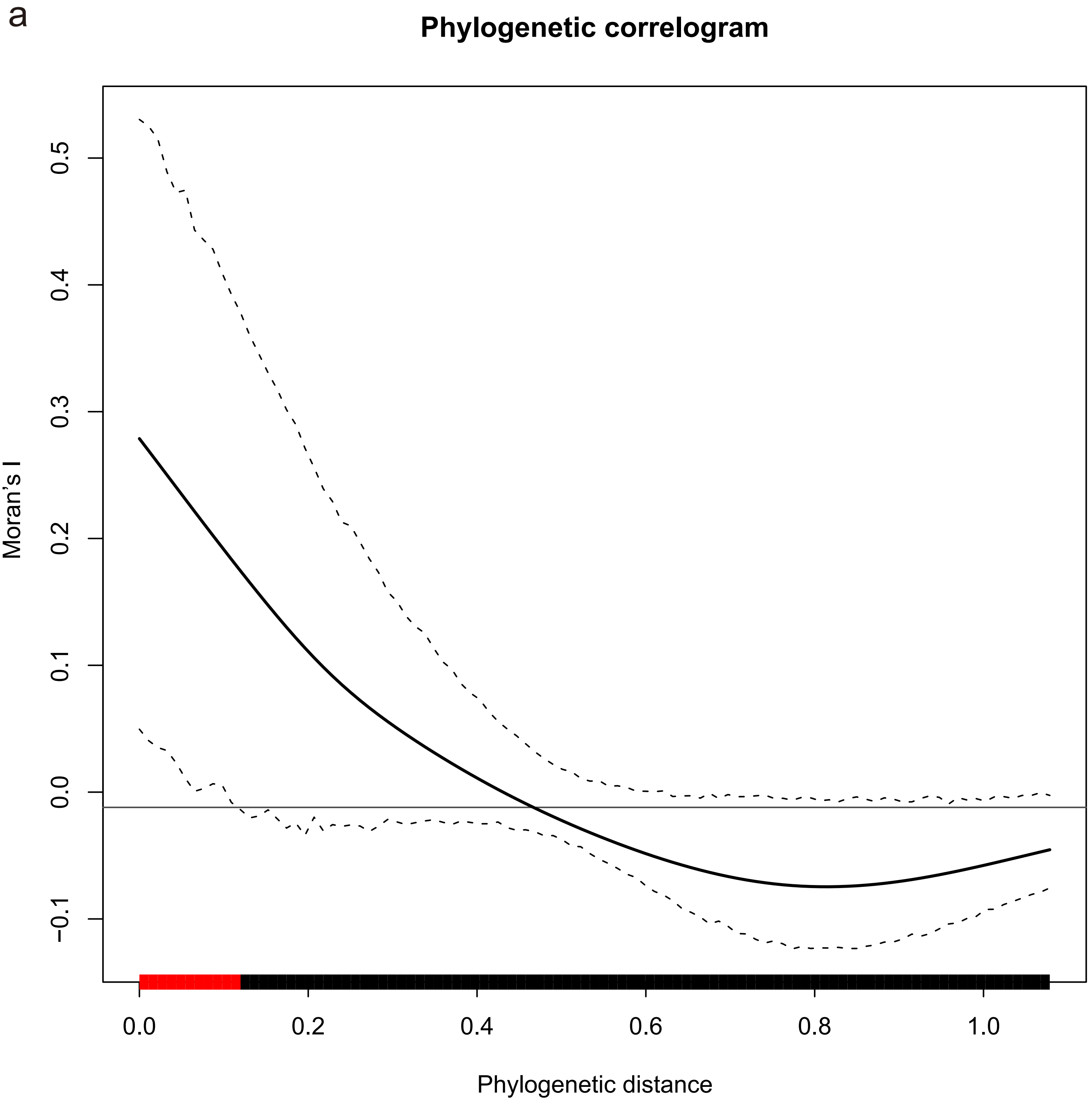

a
Phylogenetic correlogram
Moran's I
0.5
0.4
0.3
0.2
0.1
0.0
−0.1
0.0
0.2
0.4
0.6
0.8
1.0
Phylogenetic distance

b

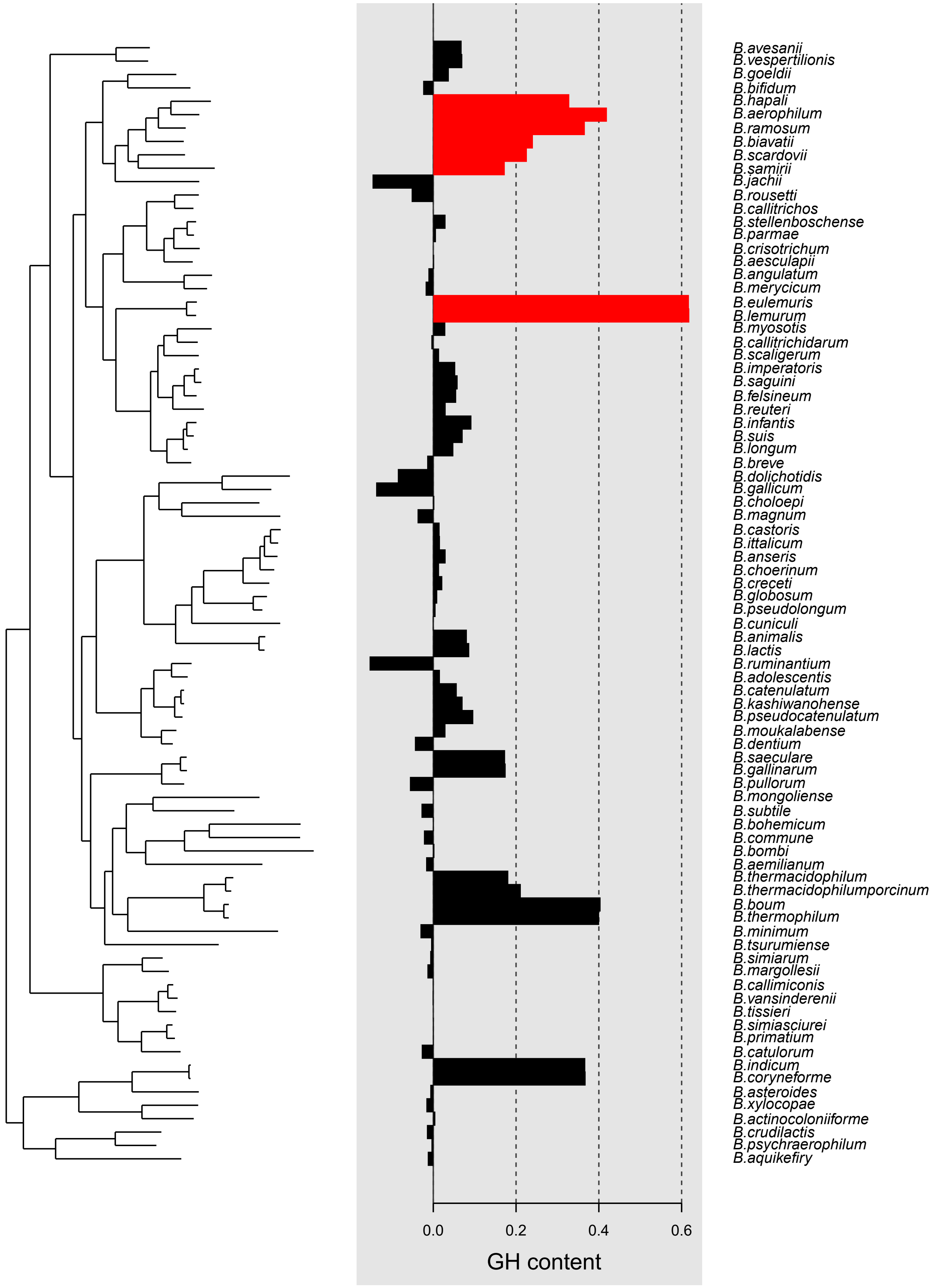

Figure S3. (a) Phylogenetic correlogram based on GH content. No significant autocorrelation was observed above the phylogenetic distance of 0.1. The dash lines represent the lower and upper confidence intervals and solid line represents the Moran's I index of autocorrelation. The colored horizontal bars at the bottom shows the significance of autocorrelation: red- significant positive autocorrelation, blue- significant negative autocorrelation and black- no autocorrelation; **(b)** Local Moran's index values for GH content for each type strain. The clades highlighted in red shows the presence of significant phylogenetic signal for p-value < 0.01.
