# Supplementary material for: Host-Diet Effect on the Metabolism of Bifidobacterium
Source: Genes (Basel). 2021 Apr 20;12(4):609. doi: 10.3390/genes12040609 (PMC8074910; doi:10.3390/genes12040609)
Supplement: Supplementary file 1 [file genes-12-00609-s001.zip › Supplementary Figure S4.pdf]

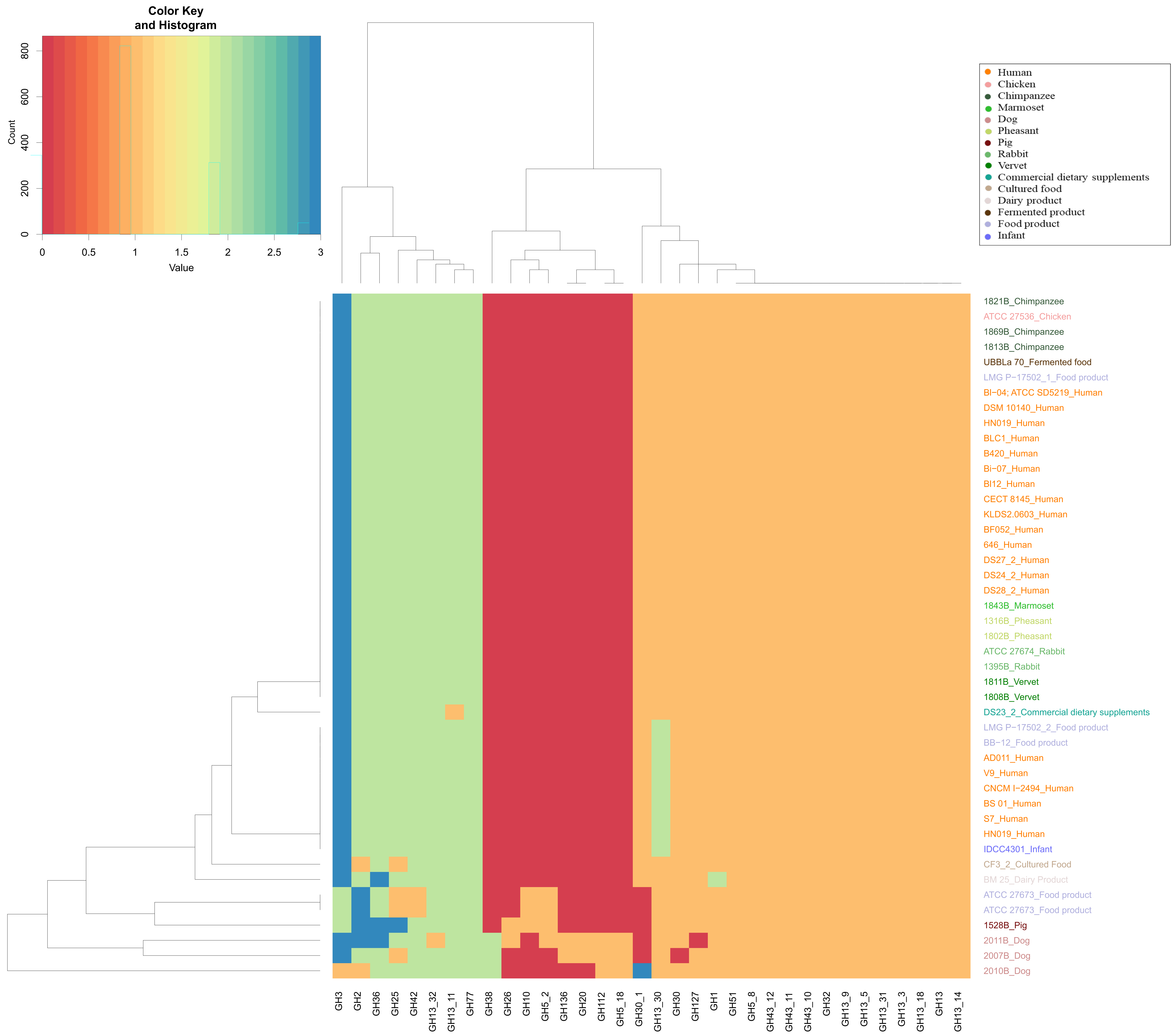

Figure S4. Clustering of *B.animalis* subsp. *lactis* strains isolated from fifteen different isolation sources. Heatmap of strains based on gene count for GHs. Strains are colored according to their isolation source as represented in the legend.
