# Supplementary material for: Host-Diet Effect on the Metabolism of Bifidobacterium
Source: Genes (Basel). 2021 Apr 20;12(4):609. doi: 10.3390/genes12040609 (PMC8074910; doi:10.3390/genes12040609)
Supplement: Supplementary file 1 [file genes-12-00609-s001.zip › Supplemenatry Table S4.docx]

**Table S4**. Selection of GH families for clustering. The chosen set is shown in bold.

| **GH Subsets** | **Sharing % in 84 taxa** | **Total GH families** | **Number of added families** | **Added families** |
| --- | --- | --- | --- | --- |
| Set 1 | 100 | 2 |  | **GH3, GH36** |
| Set 2 | 95 | 3 | 1 | **GH13** |
| Set 3 | 90 | 5 | 2 | **GH32, GH77** |
| Set 4 | 85 | 8 | 3 | **GH2, GH25, GH42** |
| Set 5 | 80 | 10 | 2 | **GH31, GH43** |
| Set 6 | 75 | 11 | 1 | **GH51** |
| Set 7 | 70 | 12 | 1 | **GH1** |
| Set 8 | 65 | 14 | 2 | **GH5, GH30** |
| Set 9 | 60 | 14 | 0 |  |
| Set 10 | 55 | 15 | 1 | **GH127** |
| Set 11 | 50 | 16 | 1 | **GH20** |
| Set 12 | 45 | 17 | 1 | **GH29** |
| Set 13 | 40 | 19 | 2 | **GH78, GH112** |
| Set 14 | 35 | 19 | 0 |  |
| Set 15 | 30 | 22 | 3 | **GH38, GH120, GH136** |
| Set 16 | 25 | 26 | 4 | **GH94, GH115, GH125, GH146** |
| **Set 17** | **20** | **32** | **6** | **GH95, GH129, GH59, GH26, GH35, GH28** |
| Set 18 | 15 | 37 | 5 | GH109, GH105, GH33, GH8, GH27 |
| Set 19 | 10 | 42 | 5 | GH101, GH121, GH23, GH53, GH65 |
| Set 20 | 5 | 48 | 6 | GH10, GH123, GH130, GH39, GH85, GH88 |
| Set 21 | 0 | 72 | 24 | GH106, GH110, GH113, GH140, GH141, GH142, GH151, GH154, GH16, GH18, GH4, GH49, GH50, GH55, GH63, GH67, GH73, GH79, GH76, GH84, GH89, GH91, GH92, GH93 |

**Selection of the GH classes for clustering**

For selection of the GH classes we checked the number of GHs in different set of strains sharing them. We started from the classes which are shared in all strains saying 100% and then dropping this percentage with the gap of 5% up to the classes shared by one to four strains (less than 5% strains). We defined these set of GH classes shared by a specific percentage of strains as sets named as (from set 1- set 21). For each of these sets we performed the hierarchical clustering and observed the clustering. We selected the set where we observed a constant clustering. The GH set of sharing level >20% produced the same clustering result as >15% and >10% indicating that the classification using 32~42 GH families was stable. The hierarchical clustering results for the selected set and sets around it are shown below i.e set 30, set 25, set 20 (selected set), set 15, set 10. We can see in the following clustering results that the clustering become almost constant for set 20, 15 and 10. Therefore, we selected the threshold of >20% in this analysis.

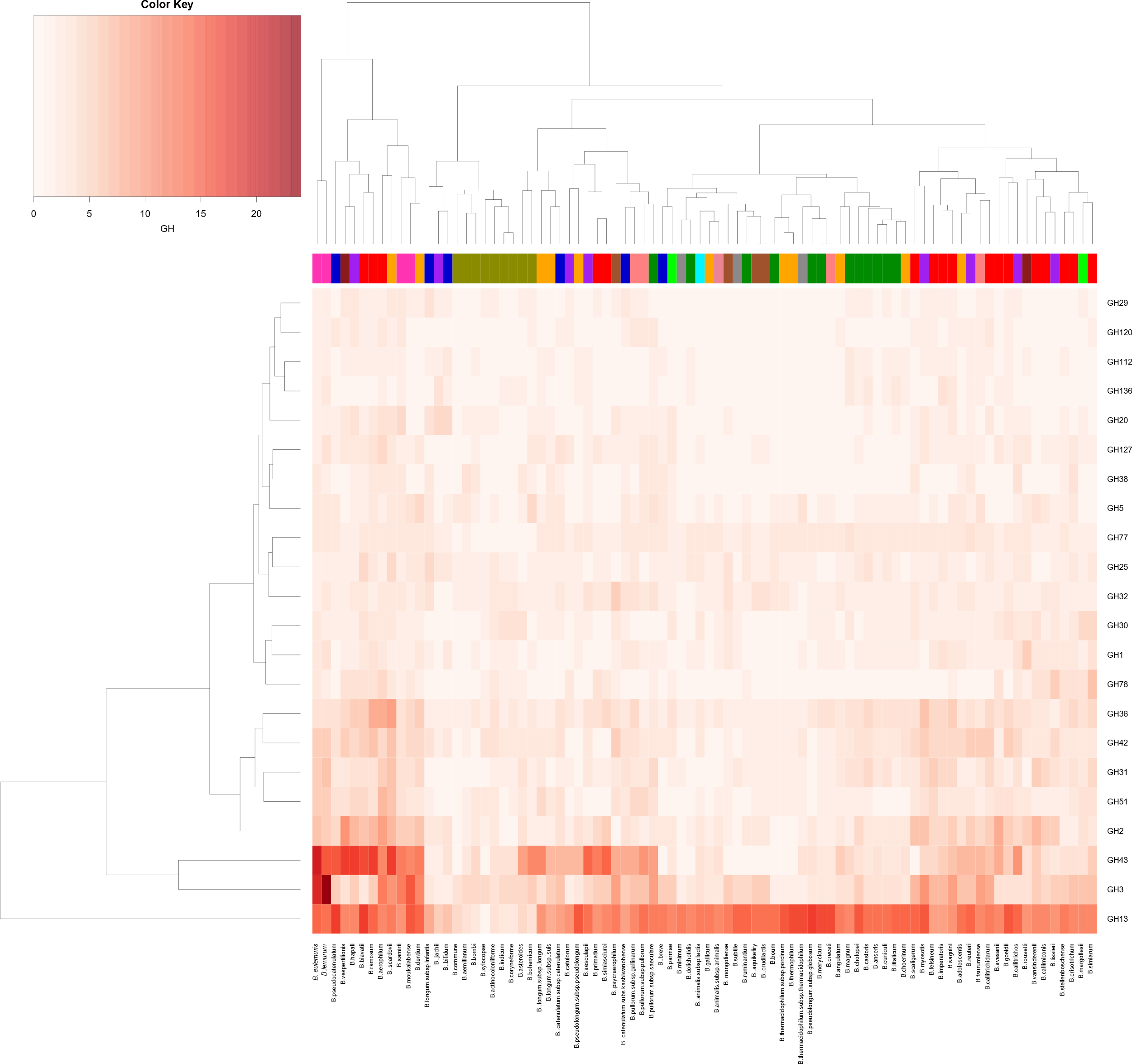


Set 30


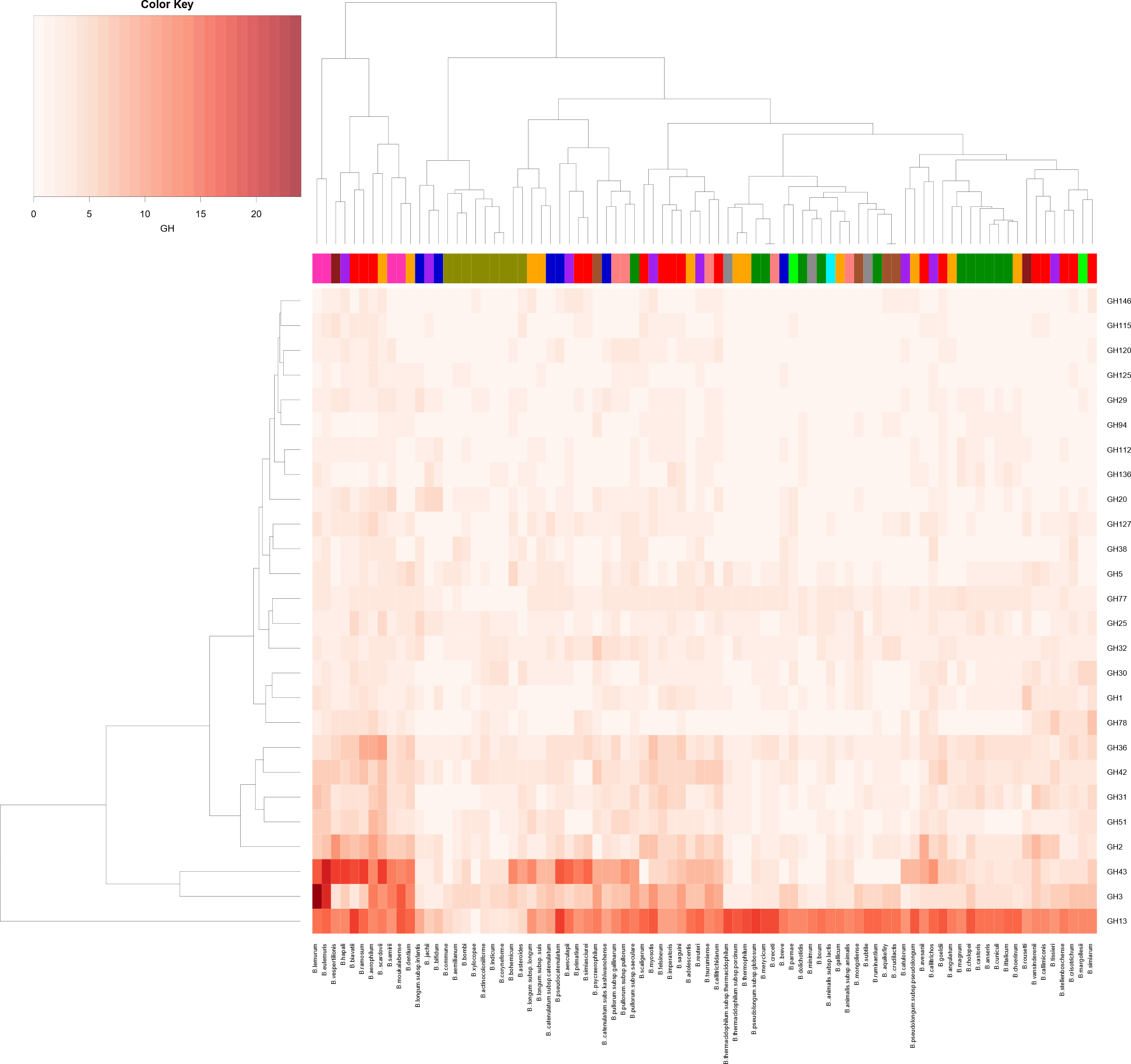


Set 25


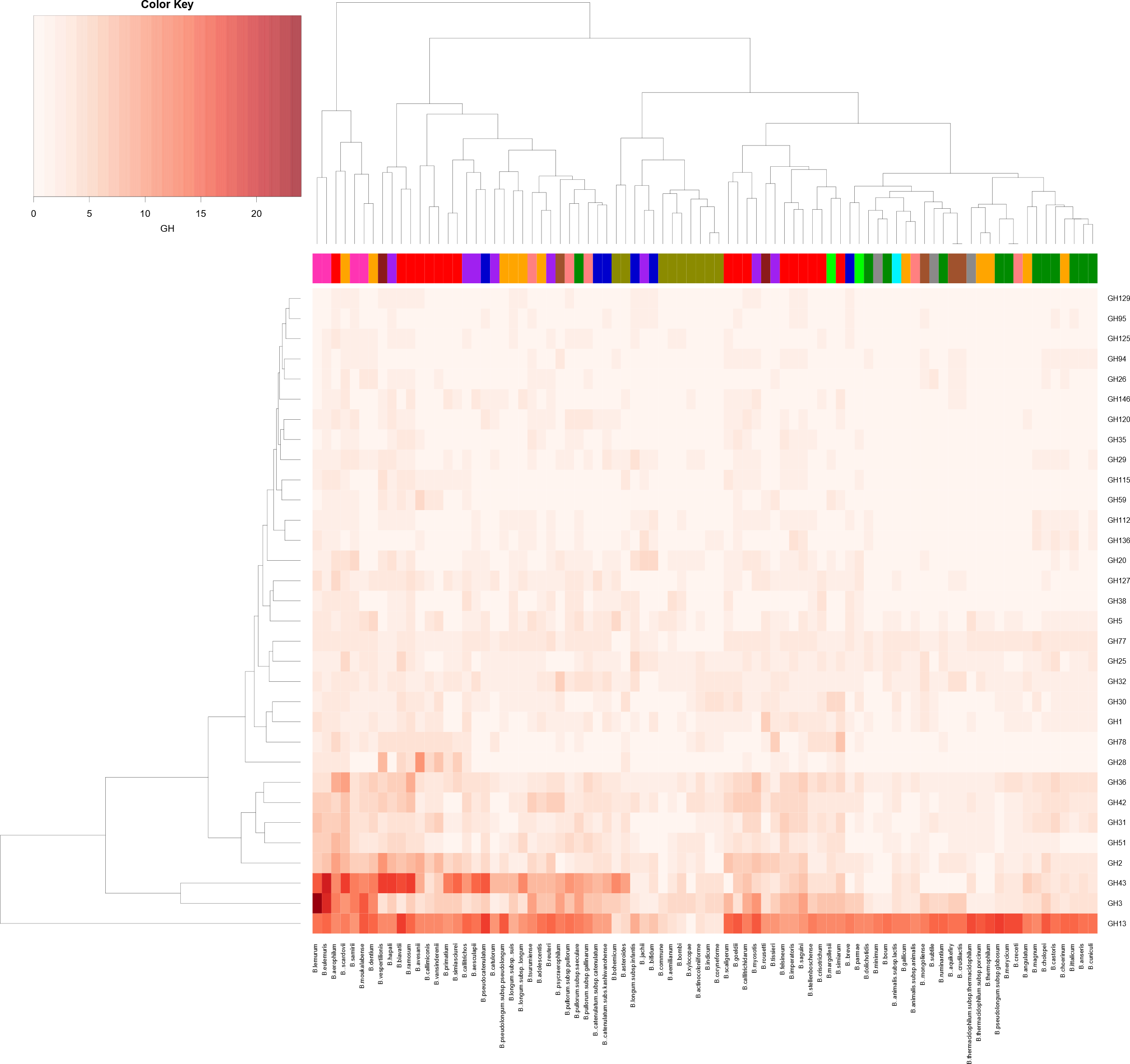


Set 20


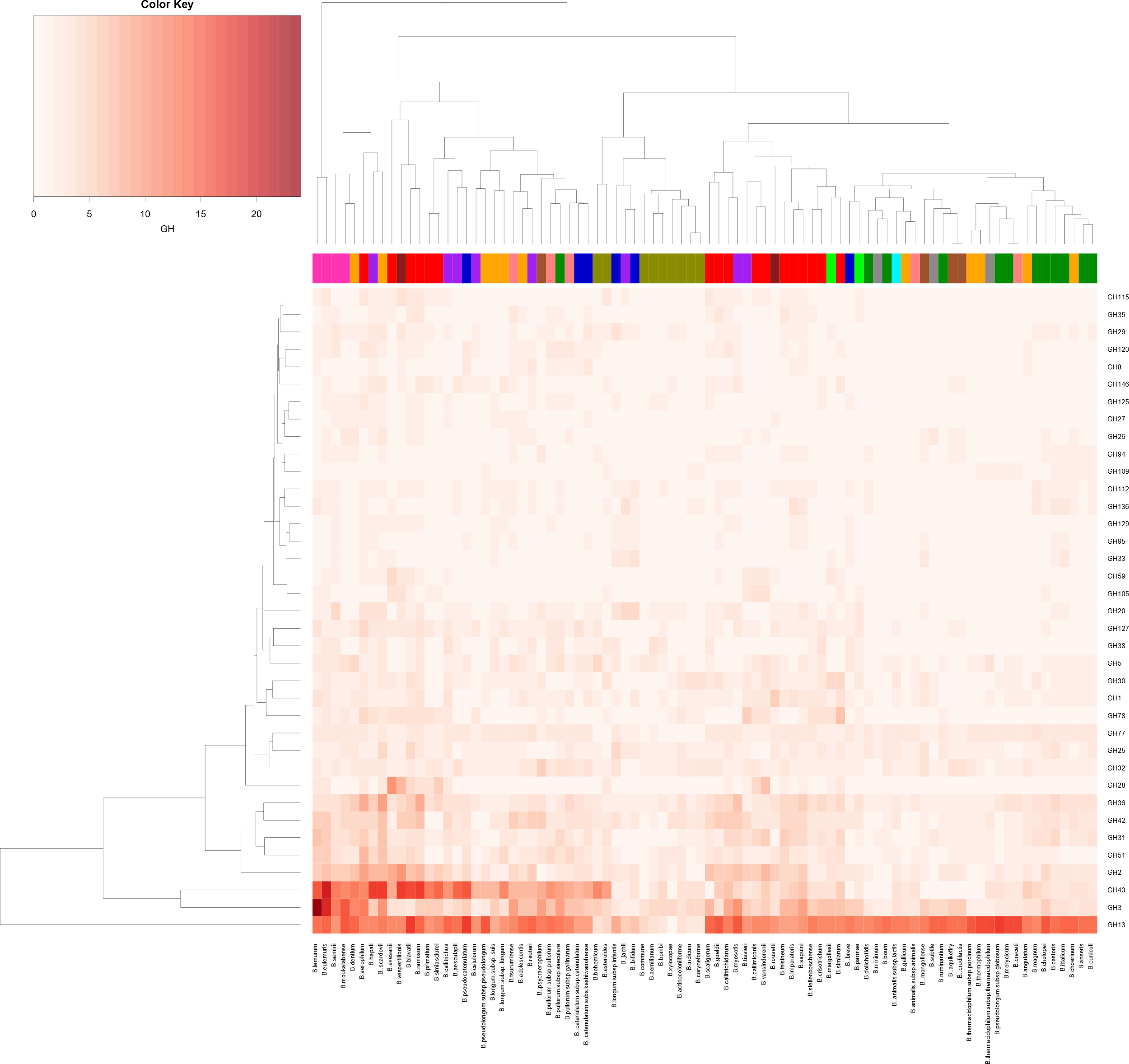


Set 15


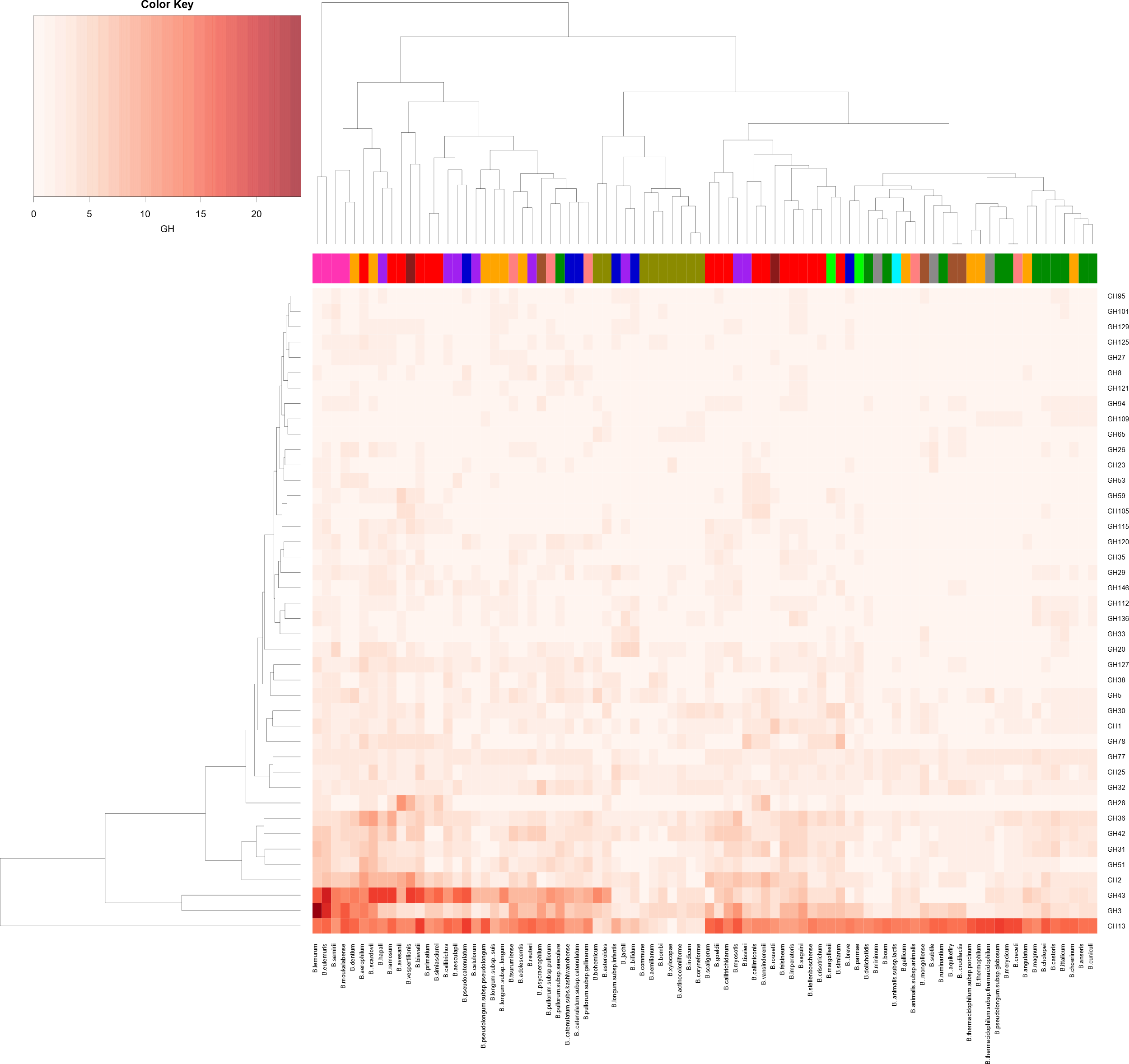


Set 10
